# Synthesis of TPGS/Curcumin Nanoparticles by Thin-Film Hydration and Evaluation of Their Anti-Colon Cancer Efficacy *In Vitro* and *In Vivo*

**DOI:** 10.3389/fphar.2019.00769

**Published:** 2019-07-12

**Authors:** Hong Li, Liping Yan, Edith K.Y. Tang, Zhen Zhang, Wei Chen, Guohao Liu, Jingxin Mo

**Affiliations:** ^1^Clinical Research Center for Neurological Diseases of Guangxi Province, The Affiliated Hospital of Guilin Medical University, Guilin, China; ^2^Department of Gastroenterology, The Second People’s Hospital of Guilin, Guilin, China; ^3^Department of Gastroenterology, The Affiliated Hospital of Guilin Medical University, Guilin, China; ^4^Department of Radiology, The Affiliated Hospital of Jilin Medical University, Jilin, China; ^5^School of Allied Health, Faculty of Health and Medical Sciences, University of Western Australia, Perth, WA, Australia; ^6^Department of Ultrasound, the First Affiliated Hospital of China Medical University, Shenyang, China

**Keywords:** curcumin, ROS, vitamin E TPGS, micellar nanoparticle, synergistic effects, colon cancer

## Abstract

Curcumin (CCM) has many potential uses in anticancer chemotherapy, but its low water solubility poses a major problem, preventing its translation into clinical use. TPGS is a water-soluble derivative of vitamin E that acts as a surfactant with the ability to form micellar nanoparticles in water. More importantly, TPGS acts as a potent antioxidant that can neutralize intracellular reactive oxygen species (ROS). In this study, we solubilized CCM with TPGS using thin-film rehydration to prepare aqueous formulations containing CCM at clinically relevant concentrations. We found that the minimal TPGS:CCM ratio for producing nanoparticles was 5:1 (w/w): at or above this ratio, stable nanoparticles formed with an average particle diameter of 12 nm. CCM was released from TPGS/CCM micelles in simulated colonic and gastric fluids. These TPGS/CCM nanoparticles were shown to decrease intracellular ROS levels and apoptosis and inhibited migration of HT-29 human colon cancer cells more potently than free CCM. Pharmacokinetic analysis showed TPGS/CCM to be more bioavailable than free CCM after oral administration to rats. Our results suggest that TPGS/CCM may increase therapeutic efficacy of CCM against colon cancer and merits further investigation in a clinical setting.

## Introduction

In recent years, there has been growing interest in the use of curcumin (CCM) for treating diseases. CCM is the active ingredient of the turmeric plant (*Curcuma longa*) and has demonstrated many chemopreventive and chemotherapeutic properties. (Scott et al., 2008; Yallapu et al., 2010) CCM has been reported to have antioxidant properties and inhibits pro-inflammatory proteins, cell proliferation, as well as tumor angiogenesis and metastasis (Nair et al., 2012). However, CCM has low water solubility, which limits oral bioavailability (Yen et al., 2010). As a result, CCM only has limited physiological effects unless very high doses are used.

One method to overcome poor aqueous solubility is to use nanoparticle techniques (Allam et al., 2015; Vecchione et al., 2016; Bagheri et al., 2018; Cheng et al., 2018). Encapsulation of CCM in nano-sized micelles enables the drug to be formulated as an aqueous dispersion at therapeutically relevant concentrations that can be administered orally with increased bioavailability and cellular uptake.

Colorectal cancer is the third most common type of cancer in the world, comprising about 10% of all cancer cases. The incidence of colorectal cancer has steadily increased over the last 25 years due to factors such as obesity (Scott et al., 2008). In 2012 alone, 1.4 million new cases of colorectal cancer were diagnosed and 694,000 deaths occurred globally (Liang and Dominitz, 2019). Colorectal cancer manifests as adenomatous polyps and malignant cells in the colon (Yallapu et al., 2010) and is one of the most aggressive cancers (Dahlhaus et al., 2018). Current screening and detection methods for colorectal cancer are also inadequate, such that most diagnoses occur at the more advanced stages of the disease when treatments are no longer effective (Dolan et al., 2018; Shimada et al., 2018; Wrobel and Ahmed, 2018).

Nanotechnology is the development and application of nanoparticles, defined as particles ranging from 10 to 100 nm, for pharmaceutical purposes (Chen et al., 2017; Mo et al., 2017; Wang et al., 2018; Zambrano et al., 2018). Nanosuspensions, nanospheres, polymeric nanoparticles, liposomes, microemulsions, and microsomes have all been developed to enhance drug delivery by modifying the rate of drug release, circulation half-life, and targeting of the drug to specific cells or tissues (Chen et al., 2018; Ni et al., 2018). Nanoparticles have been synthesized from various materials. The small size of these micellar systems and their hydrophilic interface with blood plasma components allow them to evade uptake by the reticuloendothelial system (RES) and therefore remain longer in circulation (Sharma et al., 2017). While the hydrophobic core of these micelles provides a pocket in which poorly water-soluble drugs can be dissolved, the hydrophilic shell allows the micelles to remain stably dispersed in aqueous media. This acts as a physical barrier to reduce interaction between the drug cargo and blood components or non-target cells (Huang et al., 2017).

One technique used to produce polymeric nanoparticles is the thin-film rehydration method (Chen et al., 2017). This process uses amphiphilic surfactant molecules to stabilize the nanoparticles in aqueous media. One such surfactant is polyvinyl alcohol (PVA), which has been used extensively to produce polymer nanoparticles (Ghaffari et al., 2018). However, PVA is difficult to remove completely from the final nanoparticle product, and residual PVA may cause unwanted side effects when used in health care products. For these reasons, the preferred materials for biomedical nanoparticles are natural surfactants such as cholesterol, polysaccharides, phospholipids, and vitamins.

Our laboratory has previously synthesized stable phosphate calixarene-based micellar formulations of CCM using the thin-film rehydration method (Chen et al., 2017). In the present study, we build on these efforts to formulate nanoparticles with hydrophobic drugs by replacing Pluronic F127 with d-α-tocopheryl polyethylene glycol 1000 succinate (TPGS). TPGS is a water-soluble derivative of natural vitamin E that is produced by esterifying vitamin E succinate with polyethylene glycol 1000 (Zou and Gu, 2013; Gaonkar et al., 2017). The TPGS molecule is amphiphilic, with a lipophilic alkyl tail (tocopherol succinate moiety) and a hydrophilic polar head (polyethylene glycol chain). This allows TPGS to be used as a surfactant to encapsulate hydrophobic drugs into micellar structures. Given its ability to act simultaneously as a surfactant, emulsifier, solubilizer, absorption enhancer, and antioxidant (Zhang et al., 2015). TPGS has been studied extensively in recent years as an excipient for drug delivery systems.

We hypothesized that the combined use of CCM and TPGS in a nanoparticle may have synergistic effects in the treatment of colon cancer because both CCM and TPGS can reduce levels of reactive oxygen species (ROS). We also reasoned that loading TPGS micelles with CCM (TPGS/CCM) would protect CCM from degradation in the upper digestive tract, improving CCM pharmacokinetics. Therefore, in the present study, we used the thin-film rehydration method to synthesize TPGS/CCM nanoparticles, and we evaluated their characteristics using dynamic light scattering (DLS) and UV-visible (UV-Vis) spectrophotometry. We also evaluated *in vitro* release profiles of TPGS/CCM in simulated gastric and colonic fluids and tested whether loading CCM into nanoparticles improves its anti-migratory and pro-apoptotic effects on a human colon cancer cell line. Finally, we compared the pharmacokinetic profiles of free CCM and TPGS/CCM in rats to determine whether encapsulation of the drug improves its bioavailability.

## Materials and methods

### Materials

Curcumin (purity 95.0%, C110685), tween-80, was purchased from Aladdin Chemical Reagents, Shanghai, China; TPGS was obtained from Professional Compounding Centers of America, Houston, TX, USA. Chloroform [high performance liquid chromatography (HPLC) grade] was provided by Scharlau, Barcelona, Spain and 0.2-µm filter was purchased from SARSTEDT AG & Co. KG, Nümbrecht, Germany. Methanol (analytical grade) was obtained from Univar, New South Wales, Australia. Apoptosis kits based on staining with annexin V-FITC and propidium iodide (PI) were purchased from Lianke Technology (Hangzhou, China). Emodin, which was used as internal standard was obtained from National Institutes for Food and Drug Control, Beijing, China. 35-mm glass-bottom culture dishes were purchased from NEST Biotechnology Co., Ltd. Jiangsu, China. All other materials and solvents (analytical grade) were obtained from Sigma Aldrich, St Louis, MO, USA.

### Cell Culture

HT-29 cells were obtained from Guilin UniK Biotechnology (Guilin, China) and cultured at 37.5°C in Dulbecco’s modified Eagle medium (DMEM) supplemented with 10% fetal bovine serum (FBS) and 1 mM l-glutamine. All cell culture reagents were purchased from Thermo Fisher (Pittsburgh, PA) unless otherwise mentioned.

### Animals

Male Wistar rats (200 ± 20 g) were provided by the Model Animal Research Center of Nanjing University (Nanjing, China). All animal experiments were approved by the Ethics Committee of Guilin Medical University (ethics number YXLL-2017-085).

### Preparation of TPGS/CCM Nanoparticles and Empty TPGS Micellar Particles

TPGS/CCM nanoparticles were prepared using the thin-film rehydration method developed in our laboratory. As CCM is highly hydrophobic and light-sensitive, it was handled only in glass vessels wrapped in aluminum foil. A stock solution of 10% (w/v) CCM was first prepared by dissolving 10 mg of CCM in 100 ml methanol. Solid TPGS was liquefied at 50°C, then dissolved in 50 ml HPLC-grade chloroform to produce a TPGS solution at concentrations of 0.01–1.00% (w/v) as required.

Nanoparticles were prepared at a TPGS:CCM weight ratio ranging from 50:1 to 1.5:1. For each batch of nanoparticles, relevant volumes of the CCM stock solution and the TPGS solution were mixed in a round-bottom flask, which was attached to a rotary evaporator (Eyela N1000, Japan). The solvents were evaporated at 50°C for at least 1 h or until a thin, dry film formed on the inner surface of the flask. The flask was then attached to a vacuum line for at least 12 h to dry. The dried film was rehydrated with double-deionized water that had been pre-heated to 37°C then vortexed (Vortex-Genie, New York, USA) until the thin dry film was no longer visible. We concentrated TPGS to ≥0.02% (w/v) in the final dispersion, based on the sum of masses of CCM and TPGS added at the outset. This was well above the critical micellar concentration of TPGS in water [0.02% (w/v)] (Mu and Feng, 2002). The sample was then centrifuged at 3,913 *g* for 10 min at 5°C in a refrigerated centrifuge (Sigma 2-16PK, Germany) and the supernatant was collected and filtered through a 0.2-µm filter to remove any unencapsulated CCM precipitate. The filtrate was considered a dispersion of TPGS/CCM nanoparticles, and it was lyophilized and stored in glass vials at −20°C. The nanoparticle dispersion was visually inspected for color, opacity, and presence of sediment at three stages: after rehydration of the dried film, after centrifugation, and again after filtration. TPGS/CCM samples were prepared in triplicates. Nanoparticles were resuspended in double-deionized water before use in experiments.

As blank/empty controls, TPGS micellar particles without CCM (MTPGS) were prepared using the same protocol as described above, except methanol was used instead of the CCM stock solution.

### Nanoparticle Size Determination and Stability Testing in 0.9% Saline

Nanoparticle morphology was checked using DLS following centrifugation and filtration to ensure removal of precipitated particles. DLS was performed using the Zetasizer Nanoseries (Malvern Instruments, Worcestershire, UK), and each batch was checked in quadruplicate. The morphology of MTPGS and TPGS/CCM at 12.5:1 was investigated by transmission electron microscopy (JEM-1200EX, JEOL). MTPGS and TPGS/CCM at 12.5:1 were diluted with water and then placed over 400-mesh copper-coated grids. The grids were dried at room temperature (RT) before inspection with transmission electron microscope (TEM). The UV-Vis spectra of free and TPGS-encapsulated CCM were measured at 800–200 nm using a UV-Vis spectrophotometer (Cary 50 Bio UV-visible spectrophotometer, Varian, CA, USA). Nanoparticle samples were diluted with water to give absorbances within the measurable range. A control solution of free CCM was prepared by diluting the stock solution of CCM in methanol with water.

For *in vitro* stability tests, 1 mg of freshly produced TPGS/CCM at 5:1 and 12.5:1 were incubated in 10 ml 0.9% saline at 37°C for 9 days. At different time points, the particle size distribution profiles, polydispersity index (PDI), and zeta potential values of the corresponding samples were characterized by DLS.

### *In vitro* Release Profiles of TPGS/CCM in Simulated Colonic and Gastric Fluids

*In vitro* CCM release profiles were measured using a dialysis sac in combination with a United States Pharmacopeia dissolution/release apparatus (7000/7010 Dissolution Apparatus, Agilent Technologies, Santa Clara, CA, USA). Nanoparticles were suspended in enzyme-free simulated gastric fluid (SGF) containing 0.2% tween-80 (pH 2.4) or simulated colonic fluid (SCF) containing 0.2% tween-80 and 0.13 U/ml β-galactosidase in phosphate buffer (pH 7.4). (Singh et al., 2004) The SGF was prepared by dissolving NaCl in Milli-Q (18Ω) water to a final concentration of 34.2 mM and then adjusted to pH 2.4 using 70 mM HCl (Axson et al., 2015).

TPGS/CCM sacs were dialyzed in 500 ml of either simulated fluid at 37 ± 0.5°C for 24 h with stirring at 100 cycles/min. At 0, 1, 2, 4, 8, 16, and 24 h, 0.5 ml of the release medium was sampled and the absorption at 425 nm was measured using a LAMBDA 950 UV-Vis Spectrophotometer (PerkinElmer, Wellesley, MA, USA). Each batch was analyzed in triplicates.

### Intracellular ROS Detection

Intracellular ROS generation in HT-29 cells was measured using an ROS Assay Kit. HT-29 cells were seeded in a 35-mm glass-bottom culture dishes at a density of 1 × 10^6^ cells/well and cultured overnight in 2 ml DMEM with 10% FBS at 37°C in a 5% CO_2_ humidified atmosphere. On the next day, the medium was replaced with fresh DMEM+FBS, followed by the addition of 10 µM free CCM, TPGS/CCM, or 125 µM MTPGS. Control cultures were without treatment. After 24 h, the medium was removed, 2 ml of fresh DMEM medium was added, and the mixture was incubated for another 20 min. The cells were then washed three times with ice-cold phosphate-buffered saline (PBS) and imaged under a fluorescent confocal laser scanning microscope (Leica TCS SP5, Germany). The ROS-mediated decomposition of 2,7-Dichlorodihydrofluorescein diacetate (DCFH-DA) to dichlorofluorescein (DCF) was tracked based on DCF fluorescence at an excitation wavelength of 488 nm and emission wavelengths of 500–540 nm. The mean fluorescent intensity of HT-29 cells were quantified after counting 10,000 cells by flow cytometry (BD Biosciences, Franklin Lakes, NJ, USA).

### *In vitro* Cytotoxicity

HT-29 cells were plated at a density of 1 × 10^5^ cells/well in 96-well plates. After a 24-h incubation, the culture media was replaced with 100 μl fresh media and then 100 μl formulation (TPGS/CCM, free CCM (dissolved in 0.1% DMSO), or MTPGS) was added to a final concentration of 10 or 125 µM. Control wells received 100 μl of water instead of any formulation. After incubation for 48 h, 20 μl MTT (5 mg/ml) was added to each well, the absorbance was measured at 570 nm using a UV-Vis spectrophotometer, and IC_50_ was calculated according to manufacturer protocols.

### Annexin V/PI Apoptosis Detection

HT-29 cells were seeded at a density of 1 × 10^5^ cells/well and incubated with 5 μM of MTPGS, TPGS/CCM, or free CCM for 48 h. Cells were then digested with trypsin and collected by centrifugation at 157 g for 5 min. The cells were rinsed twice with PBS, resuspended in 500 μl binding buffer, then mixed with 2.5 μl annexin V and 5 μl PI. The cells were incubated in the dark for 15 min and cell apoptosis was analyzed using flow cytometry.

### Wound Healing Assay

To investigate the effects of TPGS/CCM on cell migration, we performed a wound healing assay. Cells were seeded into six-well plates at a density of 1 × 10^6^ cells/well and cultured at 37°C in a 5% CO_2_ incubator for 24 h until completely confluent. The cell monolayer was scratched with a 200-μl pipette tip, the well was washed twice with PBS to remove any floating cells, and then the medium was replaced with DMEM containing 0.5% FBS. Cells were treated with 60 μM MTPGS, 5 μM free CCM, 5 μM TPGS/CCM, or no treatment (control) for 48 h. Migrating cells were photographed at the leading edge of the wound using a light microscope. Cell migration was quantified by dividing the area of the wound at 48 h after scratching by the area at 0 h (immediately after scratching).

### Pharmacokinetics

Male Wistar rats were fasted overnight and randomly divided into three groups (*n* = 6 per group). TPGS/CCM, MTPGS, or free CCM was then administered at a dose of 150 mg/kg by oral gavage. For the preparation of free CCM suspension, 2 ml of CCM solution (1.5 mg/ml in acetone) was added dropwise into 10 ml of 0.5% CMC-Na solution and sonicated for 0.5 h. Blood samples (0.5 ml) were collected from the suborbital vein into heparinized tubes at predetermined time points after administration. Plasma was collected by centrifugation at 10,000 *g* for 5 min and stored at –20°C.

For quantification, 200 μl plasma was mixed with 50 μl 1 mM emodin (internal standard) and then the mixture was extracted using 200 μl ethyl acetate. After vortexing for 3 min and centrifuging at 10,000 *g* for 5 min, the supernatant was transferred to a clean tube and the solvent was evaporated under nitrogen gas flow. The residue was redissolved in 200 μl mobile phase [acetonitrile: 0.5% phosphoric acid (56:44, v/v)], vortexed for 3 min and centrifuged at 10,000 *g* for 5 min. Finally, 20 μl of dissolved sample was analyzed by HPLC (Prominence, Shimadzu Corp., Japan), using a 150 × 4.6 mm Symmetry C18, 5-μm column (Kinetex, Phenomenex, CA, USA), and the mobile phase pumped at a flow rate of 1 ml/min. Pharmacokinetic parameters were calculated using the non-compartmental model in DAS 2.0 (Mathematical Pharmacology Professional Committee of China, Shanghai, China).

### Statistical Analysis

All batches were produced in triplicates, otherwise mentioned. Each experiment was repeated twice. Experimental results are presented as mean ± SD and were analyzed using ANOVA and Student’s *t* test. A *p*-value of <0.05 was considered statistically significant.

## Results

### Characterization of TPGS/CCM Nanoparticles

**Figure 1:**
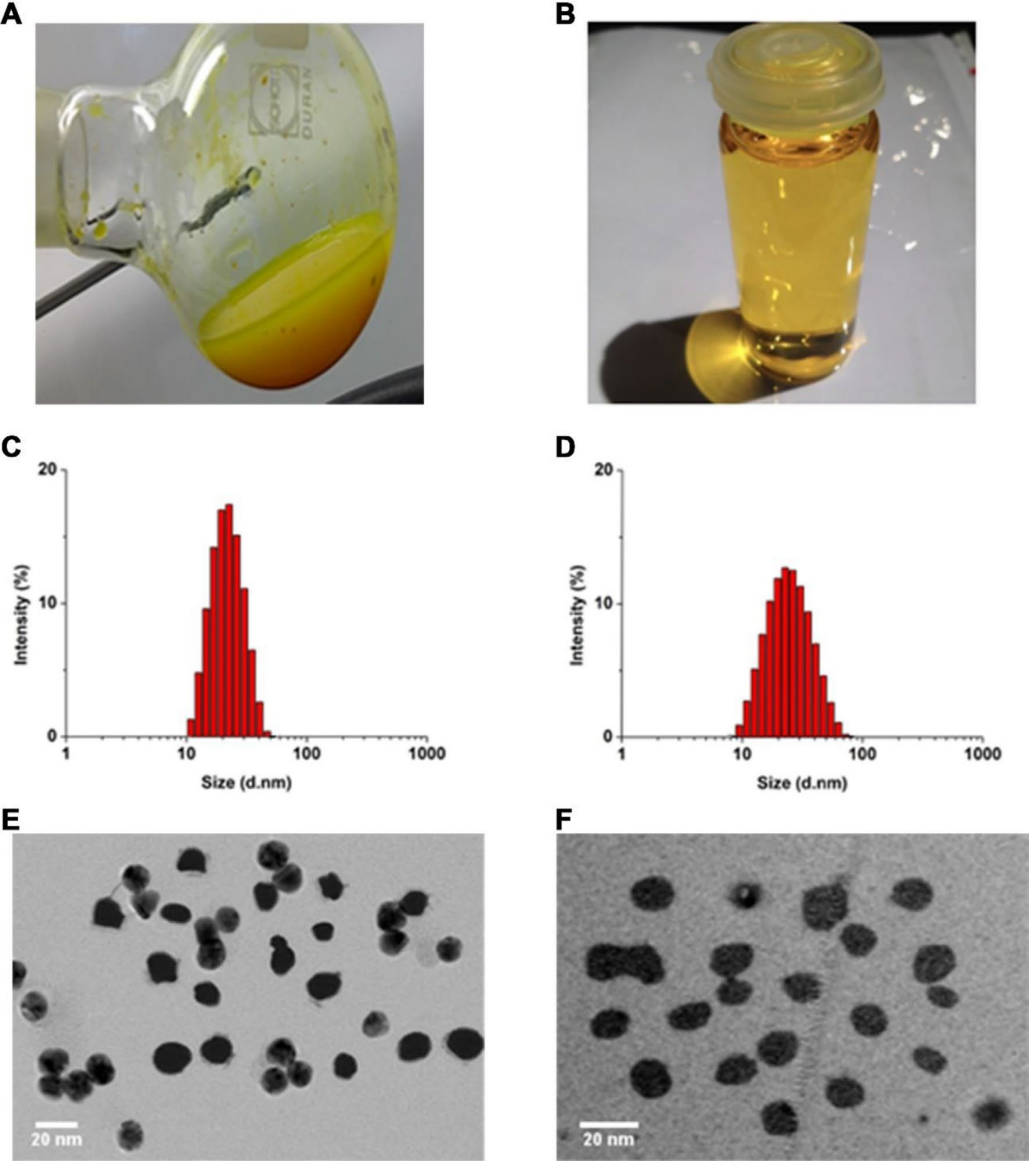
Gross appearance of nanoparticle dispersions prepared using TPGS:CCM ratios of **(A)** 3.5:1 and **(B)** 12.5:1. Sample **(A)** was translucent orange with visible precipitation, while **(B)** was transparent yellow. Size distribution of **(C)** MTPGS micelles and **(D)** 12.5:1 of TPGS/CCM micelles, based on dynamic light scattering. Transmission electron micrographs of **(E)** MTPGS micelles and **(F)** 12.5:1 of TPGS/CCM micelles. Scale bar, 20 nm.

Nanoparticle dispersions prepared with a TPGS:CCM weight ratio ≥5:1 consistently formed transparent yellow dispersions with no sediment visible (**Figure 1A**), indicating that the whole CCM load was successfully solubilized (i.e. 100% loading efficiency). These preparations remained stable based on visual inspection for at least 7 days, with stability increasing with higher TPGS:CCM ratio. In contrast, dispersions prepared with TPGS:CCM at ratios <5:1 formed a translucent orange liquid (**Figure 1B**) that yielded a sediment upon centrifugation, indicating the presence of unencapsulated, insoluble CCM.

Nanoparticle size and size distribution were examined using dynamic laser scattering. As shown in **Figures 1C and D**, loading TPGS micelles with CCM (at 12.5:1, w/w) did not visibly affect their sizes or size distribution. The average particle sizes of MTPGS and TPGS/CCM at 12.5:1 were 12.7 ± 0.1 nm (PDI = 0.18) and 12.3 ± 0.1 nm (PDI = 0.17), respectively (*n* = 3, *P* > 0.05), suggesting a narrow size distribution. Transmission electron micrographs revealed that MTPGS and TPGS/CCM (at 12.5:1) nanoparticles were spherical and their sizes are consistent with the results of dynamic light scattering (**Figures 1E and F**). The lack of change in particle size and size distribution observed due to drug loading is consistent with previous studies (Saxena and Hussain, 2012; Meng et al., 2017). One of the possible reasons for this phenomenon is that the relatively low concentration (7.58%, w/w) and low molecular weight (368.39 g/mol) of CCM has negligible impact on TPGS micelles formation.

**Figure 2:**
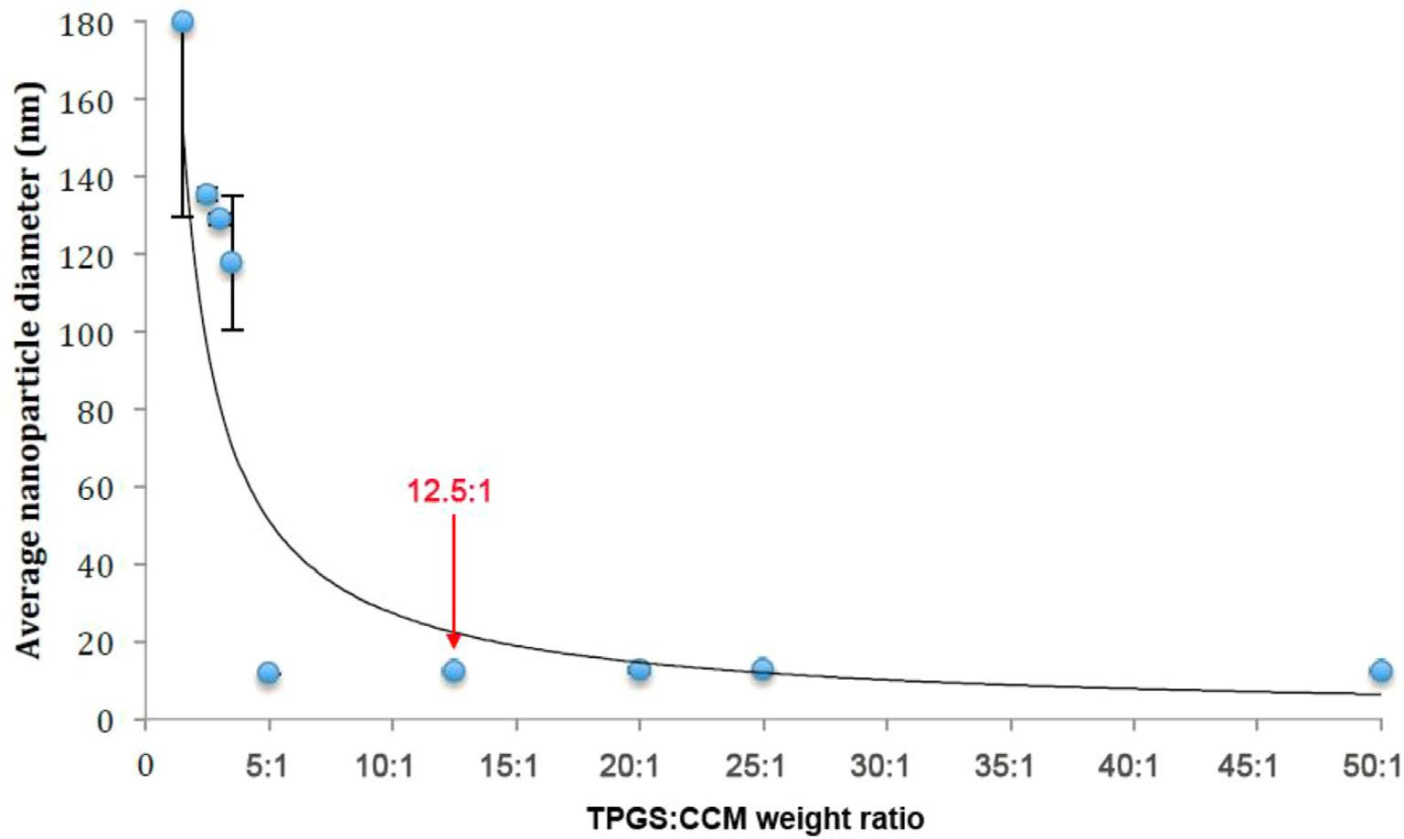
Sizes of nanoparticle prepared at different TPGS:CCM ratios.

Nanoparticle size as measured by DLS is summarized in **Table 1**. A scatterplot of particle size *versus* TPGS:CCM ratio revealed an inflexion point at a ratio of 5:1, suggesting that this was the threshold for effective nanoparticle formation (**Figure 2**). Nanoparticles formed at TPGS:CCM ratios ≥5:1 were smaller, with a mean size of 11.6–12.7 nm. These sizes were comparable to that of MTPGS (12.7 nm). In contrast, nanoparticles formed at TPGS:CCM ratios <5:1 were about 10-fold larger (117.6–179.9 nm).

**Table 1:** Nanoparticle morphology, size, and stability of nanoparticle dispersions at different TPGS:CCM ratios.

TPGS:CCM ratio (w/w)	Size, mean ± SD (nm, *n* = 8)	Polydispersity index	Color	Other observations
*1:0 (blank)	12.7 ± 0.1	0.18	Colorless	Transparent
50:1	12.3 ± 0.1	0.15	Yellow	Transparent
25:1	12.7 ± 0.1	0.18	Yellow	Transparent
20:1	12.5 ± 0.1	0.22	Yellow	Transparent
12.5:1	12.3 ± 0.1	0.17	Yellow	Transparent
5:1	11.6 ± 0.2	0.16	Yellow	Transparent
3.5:1	117.6 ± 17.2	0.28	Orange	Translucent; sediment upon centrifugation
3:1	129.0 ± 1.5	0.17	Orange	Translucent; sediment upon centrifugation
2.5:1	135.4 ± 1.6	0.12	Orange	Translucent; sediment upon centrifugation
1.5:1	179.9 ± 50.3	0.38	Orange	Translucent; sediment upon centrifugation

### Stability of Nanoparticles at Different TPGS:CCM Ratios in 0.9% Saline

**Figure 3:**
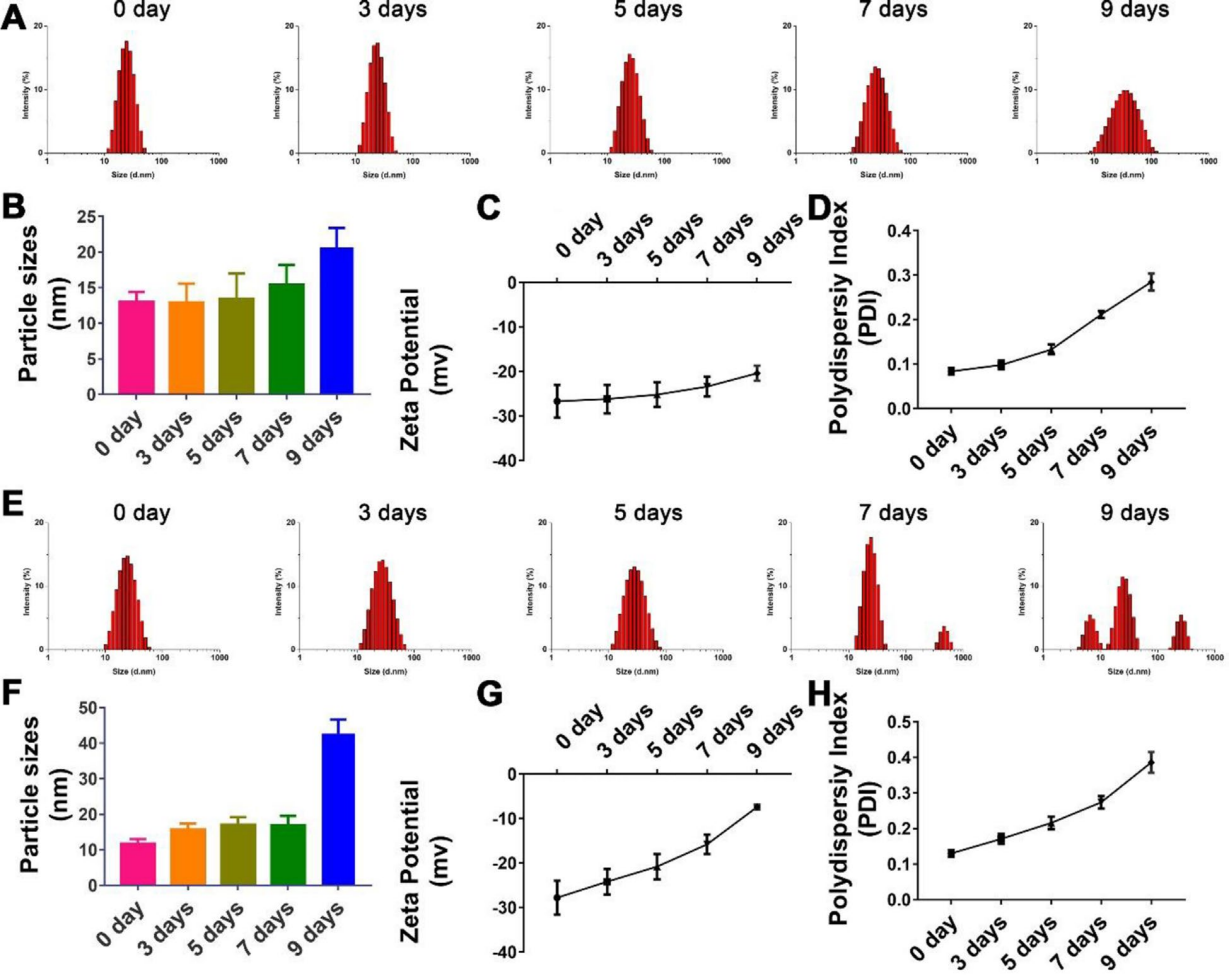
Stability of nanoparticle with TPGS:CCM ratios at **(A–D)** 12.5:1 and **(E–H)** 5:1 in 0.9% saline solution after 9 days. Changes in size distribution profiles of **(A)** 12.5:1 and **(E)** 5:1, and mean diameter of **(B)** 12.5:1 and **(F)** 5:1; zeta potential of **(C)** 12.5:1 and **(G)** 5:1 and polydispersity index (PDI) of **(D)** 12.5:1 and **(H)** 5:1 nanoparticle dispersions in 0.9% saline for up to 9 days. Results are mean ± SD (*n* = 3).

As shown in **Figure 3A and B**, though there were no significant changes in the mean particle size upon incubation of the TPGS/CCM at weight ratio of 12.5:1 in 0.9% saline for up to 7 days (*p* > 0.05), the size distribution widened. Moreover, the PDI and zeta potential increased with extended incubation times (**Figures 3C, D**), indicating that the TPGS/CCM at 12.5:1 were not stable beyond 7 days and required preparation at the time of use or lyophilization for storage.

The hydrolytic stability of TPGS/CCM at weight ratio of 5:1 was shown to be poorer than that of TPGS/CCM at 12.5:1 (**Figure 3**). After 9 days of incubation in 0.9% saline, the TPGS/CCM at 5:1 aggregated into bigger particles and formed multiple peaks due to a sharp decrease in absolute value of zeta potential. In contrast, the size distribution of TPGS/CCM at 12.5:1 remained narrow, indicating higher hydrolytic stability under the same conditions. Thus, a TPGS:CCM ratio of 12.5:1 was chosen to be used in this study, because of the relatively higher stability of the resulting drug-loaded nanoparticles.

**Figure 4:**
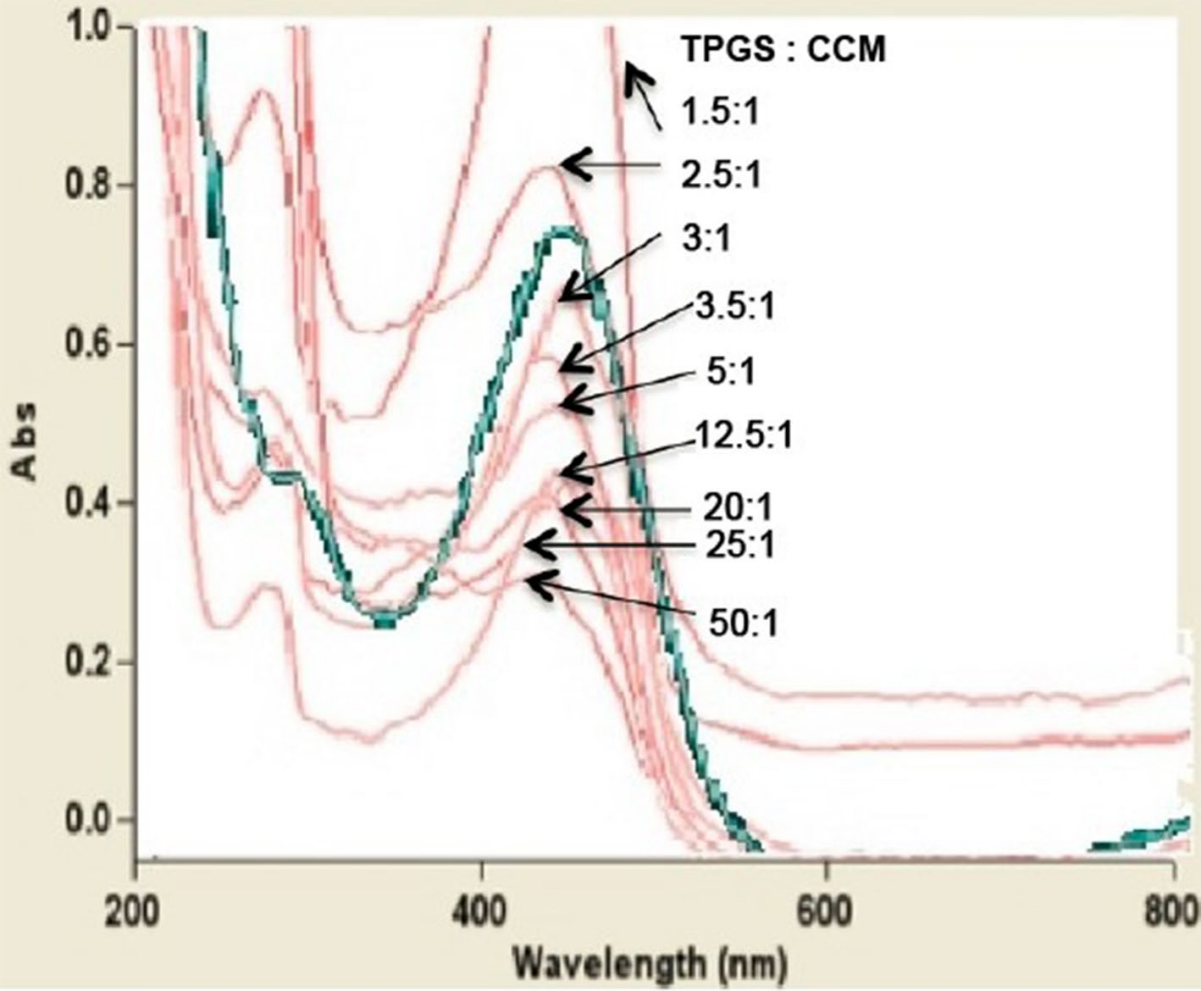
UV-visible spectra for nanoparticle dispersions prepared at different TPGS:CCM ratios. The spectrum for free CCM is shown as a green line.

All nanoparticle dispersions showed an absorption peak at 425 nm, which corresponds to the absorption peak of free CCM (**Figure 4**). This suggests that the chemical structure of CCM was retained regardless of the TPGS:CCM ratio used to prepare the samples, and that there was no chemical reaction between CCM and TPGS. This is consistent with our hypothesis that the hydrophobic CCM is physically trapped within the core of the TPGS micelles. Absorbance at this peak wavelength changed with CCM concentration in accordance with the Beer–Lambert Law.

### TPGS/CCM Nanoparticles Release CCM More Effectively in Simulated Colonic Fluid Than Simulated Gastric Fluid

**Figure 5:**
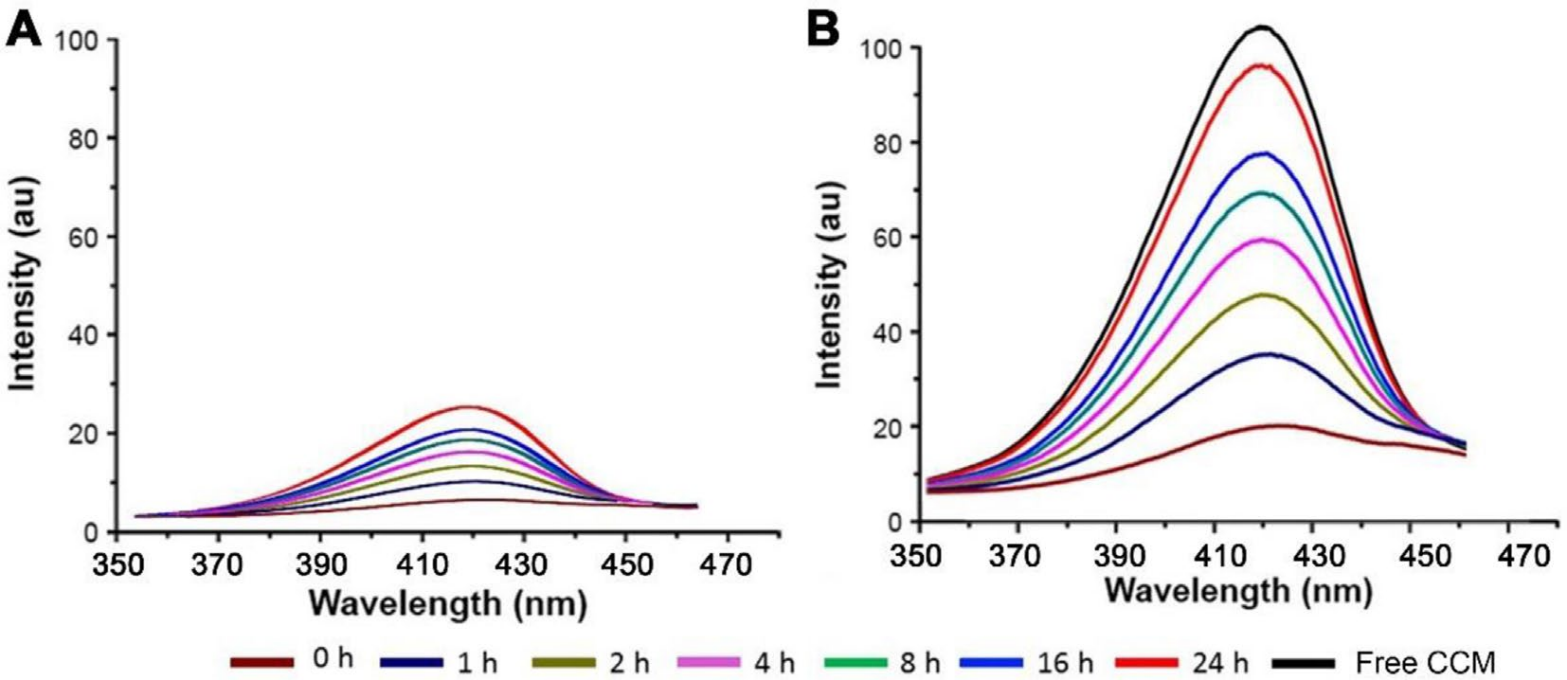
Fluorescence spectra of CCM released from TPGS/CCM in **(A)** simulated gastric fluid and **(B)** simulated colonic fluid at different time points. The spectrum of free CCM is shown as a black line.

*In vitro* CCM release profiles of TPGS/CCM in each type of simulated fluid are shown in **Figure 5A and B**. The total amount of CCM released was lower in SGF than in SCF, with only about 25% of initial CCM released in gastric fluid after 24 h at 37°C. In contrast, more than 40% of initial CCM was released in colonic fluid within only 2 h and 95% was released by 24 h. We suggest that the greater release in colonic fluid may reflect oxidation of TPGS by β-galactosidase, which destabilizes the nanoparticles and thereby accelerates CCM release.

### TPGS/CCM Nanoparticles Reduce the Intracellular Concentration of ROS in HT-29 Cells

**Figure 6:**
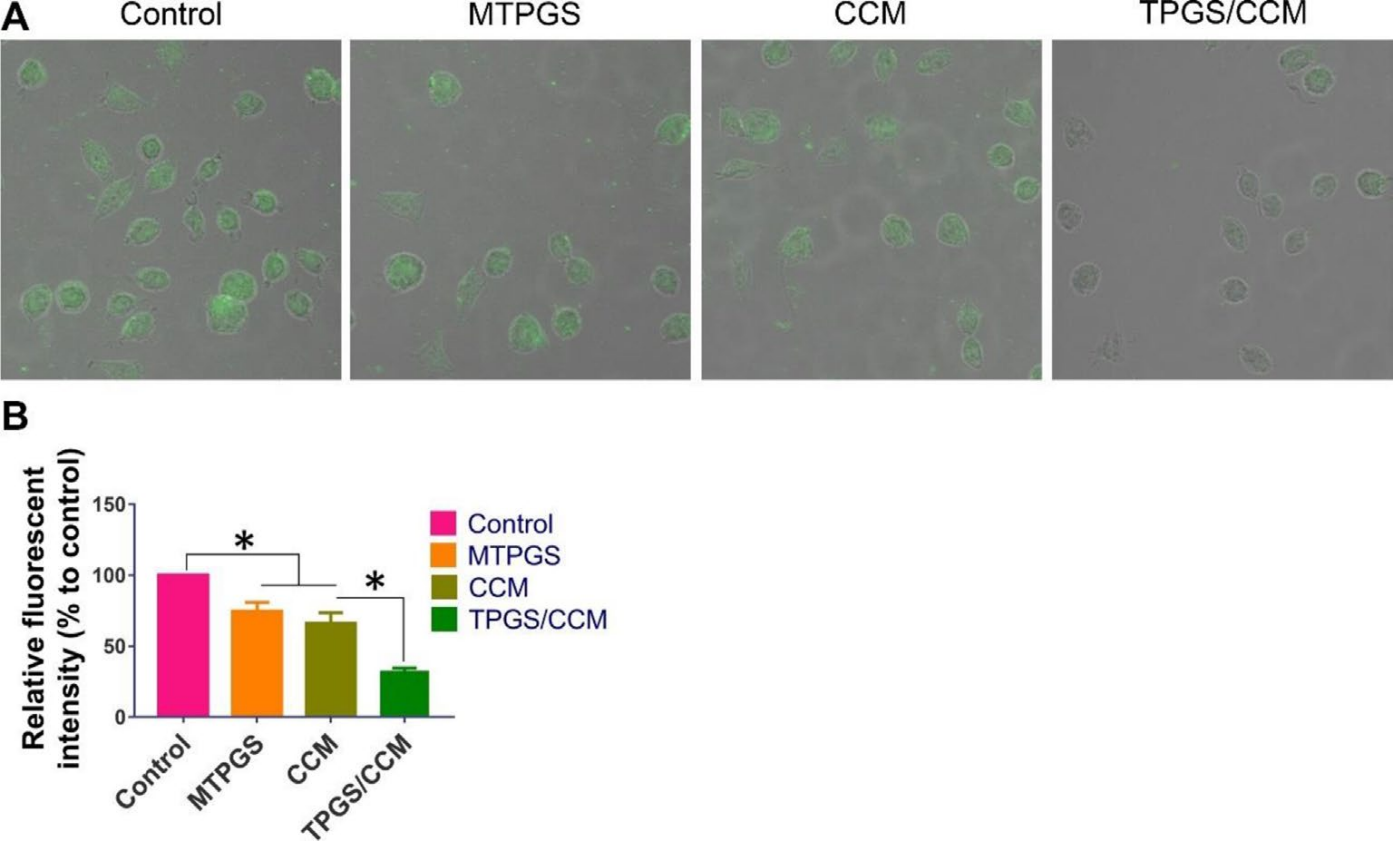
DCF fluorescence in HT-29 cells treated with TPGS/CCM. **(A)** Representative images of cells treated with TPGS/CCM, MTPGS, free CCM, or no treatment (control). DCF fluorescence is shown in green overlaid on the bright-field image. Magnification, 500X. **(B)** Quantitation of DCF fluorescence in treated cells as a percentage of fluorescence in control cells. *p < 0.05.

Treatment of HT-29 cells with blank MTPGS or free CCM alone significantly decreased DCF fluorescence intensity compared to control cells, indicating a reduction in the level of intracellular ROS (**Figure 6**). Furthermore, DCF fluorescence was significantly lower in HT-29 cells treated with TPGS/CCM than in cells treated with free CCM or MTPGS.

### Cytotoxicity of the TPGS/CCM Against HT-29 Cells

**Figure 7:**
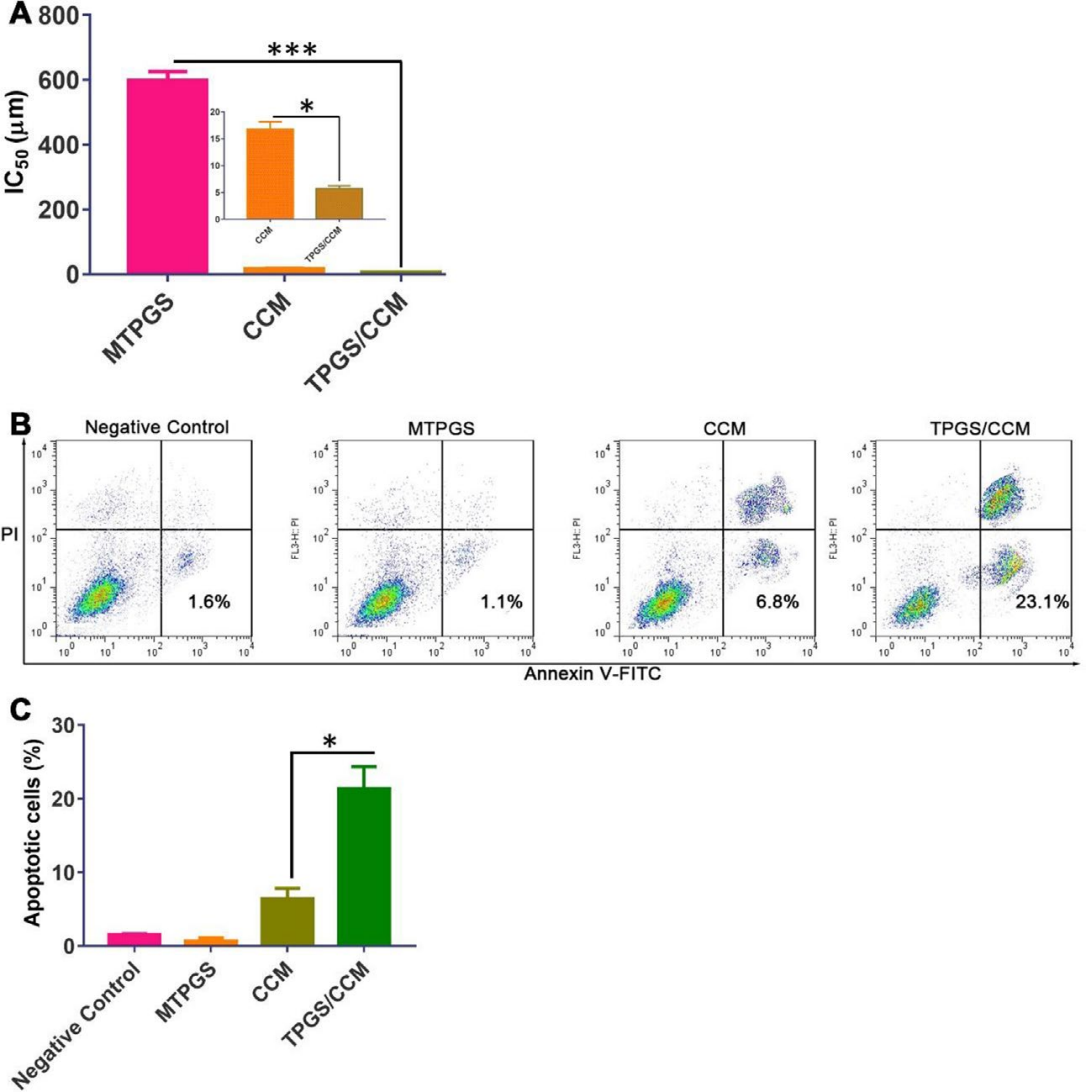
HT-29 cell viability after treatment with TPGS/CCM, MTPGS, or free CCM. **(A)** Cytotoxicity was measured using the MTT assay. The inset in **(A)** shows a magnified view of the CCM and TPGS/CCM groups. **(B)** Flow cytometric analysis of HT-29 cells treated with different formulations. **(C)** Percentage of apoptotic cells in different treatment groups. *p < 0.05, ***p < 0.001.

HT-29 cells treated with TPGS/CCM had a significantly lower IC_50_ (5.7 ± 0.5 μM) than those treated with free CCM (16.8 ± 1.4 μM), which in turn had a significantly lower IC_50_ than cells treated with MTPGS (598.7 ± 27.4 μM) (**Figure 7A**). These results indicate that TPGS/CCM is more efficient at lower doses than free CCM alone.

### Effects of CPM on BT-549 Apoptosis

The apoptosis results reflected by the flow cytometric analysis are shown in **Figure 7B**. Treatment of HT-29 cells with 5 μM free CCM resulted in 6.47 ± 1.4% annexin V-positive cells while cells treated with 5 μM TPGS/CCM resulted in 21.4 ± 3.0%. In other words, loading CCM into TPGS nanoparticles resulted in a 3.3-fold increase in HT-29 cell apoptosis. The collective data shown in **Figure 7C** suggests that CCM loaded in TPGS had evident superiority as compared with the free CCM or the carrier alone.

### TPGS/CCM is More Effective Than Free CCM at Reducing Colon Cancer Cell Migration

**Figure 8:**
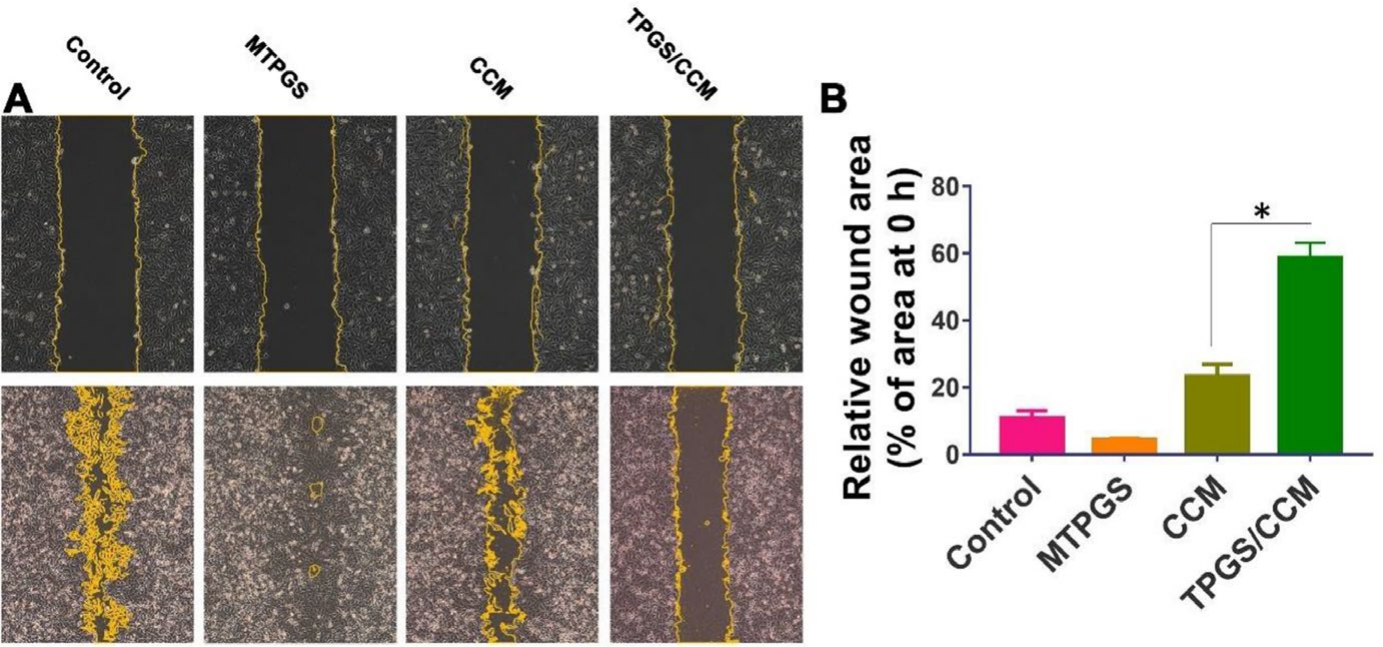
Wound healing assay in HT-29 cells treated for 48 h with TPGS/CCM, MTPGS, free CCM, or no treatment (control). **(A)** Phase-contrast micrographs of the wound area at 0 h (upper row) and 48 h (lower row) later. Magnification, 100X. Cell edges along the scratch are falsely colored brown for visibility. **(B)** Quantitation of the wound area in each treatment group at 48 h, expressed as a percentage relative to the wound size at time 0. *p < 0.05.

After 48 h of incubation, the HT-29 cells in the control group had migrated to nearly completely cover the wound area, as had cells treated with MTPGS (**Figure 8A**). In contrast, migration was markedly reduced in the CCM-treated groups. Cells treated with free CCM had a wound area that was 23.2 ± 4.1% of the original size, while cells treated with TPGS/CCM showed a wound area that was 58.8 ± 4.3% of the original size (**Figure 8B**).

### Pharmacokinetic Profiles of Orally Administered TPGS/CCM

**Figure 9:**
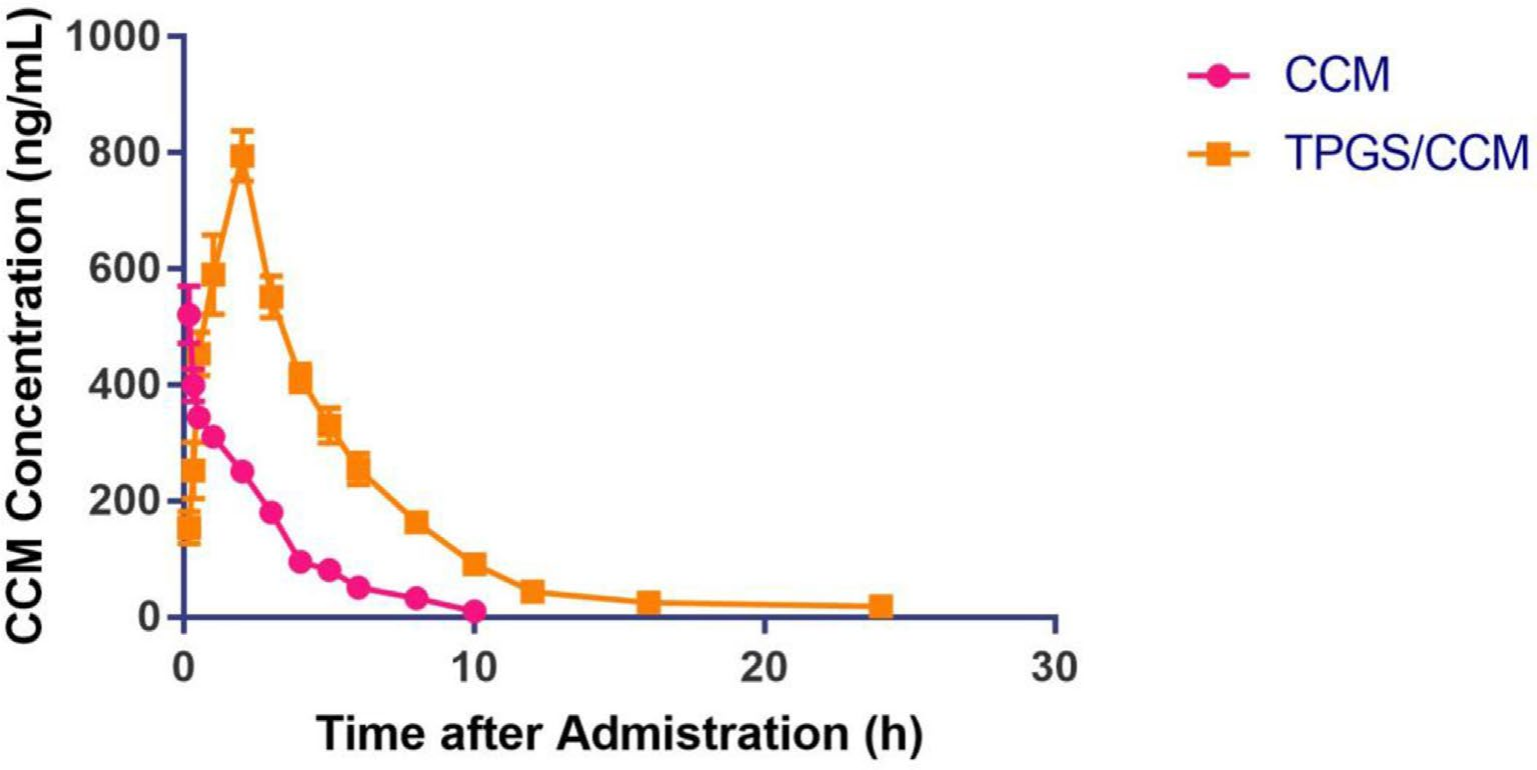
Mean plasma CCM concentrations after oral administration of either free CCM or TPGS/CCM in rats (*n* = 6 per group).

The pharmacokinetic parameters of free CCM and TPGS/CCM are summarized in **Table 2** and the CCM blood concentration shown in **Figure 9**. For free CCM, the maximum drug concentration (*C*_max_) achieved was 311.42 ± 15.51 ng/ml, which occurred immediately after administration (*T*_max_ = 0). In contrast, the *C*_max_ of TPGS/CCM was substantially higher at 794.97 ± 43.94 ng/ml, and occurred much later at 2 h after administration. The AUC_0→_ *_24_* of TPGS/CCM was 3,461.48 ± 102.47 ng/ml/h, nearly 6.5-fold higher than that of free CCM (529.49 ± 22.32 ng/ml/h). These results indicate that loading CCM into TPGS nanoparticles significantly enhances its systemic absorption, increasing its C_max_ and producing a more sustained release profile.

**Table 2:** Pharmacokinetic parameters of free curcumin (CCM) and TPGS/CCM.

Parameter	Free CCM	TPGS/CCM
C_max_ (ng/ml)	311.42 ± 15.51	794.97 ± 43.94
T_max_ (h)	0	2
T_1/2_ (h)	0.35 ± 0.07	3.16 ± 0.78
Ke (h^−1^)	0.133	0.133
AUC_0–24_ (ng • h/ml)	529.49 ± 22.32	3,461.48 ± 102.47

## Discussion

Loading lipophilic drugs into nanoparticles renders them dispersible in water (Bosselmann and Iii, 2012). CCM has great therapeutic potential but is poorly soluble in water (Nair et al., 2012). In this study, we demonstrate a method for loading CCM into nanoparticles using the surfactant, TPGS.

Optimizing the surfactant:drug ratio in nanoparticles is important for ensuring that their dispersions show low viscosity, high stability, and minimal particle aggregation (Tang et al., 2013). We found that the ratio of TPGS:CCM used was critical to the formulation of TPGS/CCM nanoparticles where stable dispersions were observed only for nanoparticles formulated at ratios >5:1. UV spectrophotometry analysis showed that the chemical structure of CCM was unchanged after encapsulation in TPGS/CCM nanoparticles. This suggests that CCM maintains its chemical properties and therefore its pharmacological activities within the TPGS micelles.

Two issues arose when dispersions were prepared using TPGS:CCM ratios below 5:1. First, precipitation was observed, even when the concentration of TPGS was well above its critical micellar concentration. This suggests that TPGS micellar volume could not accommodate the CCM load, leading to precipitation of excess CCM. Second, the nanoparticles formed were much larger than blank MTPGS micelles. We interpret this to mean that these dispersions did not contain TPGS micellar structures. The increase in particle size may also be indicative of decreasing nanoparticle stability, although the relatively low polydispersity indices for these dispersions suggests a narrow particle size range. These results, together with the observation that nanoparticles >100 nm are unable to enter cells by receptor-mediated processes (Montes-Burgos et al., 2010), leads us to recommend formulating TPGS/CCM nanoparticles at TPGS:CCM ratios >5:1.

We found that loading CCM into TPGS/CCM nanoparticles significantly improved the effects of the drug on HT-29 cells *in vitro*. ROS are known to be a key determinant of metabolic phenotype in cancer cells. Studies have shown that cancer cells have higher steady-state ROS levels than normal cells, and treating cancer cells with antioxidants can prevent their proliferation, invasion, migration, and metastasis (Nishikawa et al., 2009; Subramani et al., 2016). In our study, neither free CCM nor blank MTPGS affected intracellular ROS levels, whereas TPGS/CCM nanoparticles effectively reduced these levels. Both TPGS and CCM individually are known to have antioxidant properties at their rather high concentrations. One possible reason that only TPGS/CCM strongly reduced ROS levels is that TPGS and CCM simultaneously enter single cell and act synergistically to neutralize ROS.

TPGS/CCM was also more effective than free CCM at inducing apoptosis and inhibiting cell migration of HT-29 cells. One possibility is that loading into nanoparticles allows CCM to enter the cells more effectively by endocytosis and act synergistically with TPGS. Together, these results indicate that packaging CCM into TPGS nanoparticles increases drug potency.

Pharmacokinetic assessment of orally administered TPGS/CCM shows that the absorption modes of free CCM and TPGS/CCM are not the same, which is consistent with *in vitro* results. Loading CCM into TPGS/CCM nanoparticles provides greater drug bioavailability than free CCM when orally administered, which may mean that lower doses can be used. In other words, loading CCM into nanoparticles that are readily dispersible in aqueous media may allow administration of clinically relevant CCM doses by any route.

## Conclusion

We showed that the thin-film rehydration method can be used to produce water-soluble CCM-loaded TPGS micellar nanoparticles. These nanoparticles efficiently release CCM in simulated colonic fluid and are significantly more effective than free CCM at reducing ROS concentration, increasing apoptosis, and inhibiting migration of HT-29 colon cancer cells *in vitro*. We also showed that CCM orally administered to rats is more bioavailable when formulated as TPGS/CCM than free CCM. These TPGS/CCM nanoparticles may therefore form the basis for the development of novel CCM formulations for treatment of colon cancer.

## Ethics Statement

Male Wistar rats (200; 20 g) were provided by the Model Animal Research Center of Nanjing University (Nanjing, China). All animal experiments were approved by the Ethics Committee of Guilin Medical University (ethics number YXLL-2017-085).

## Author Contributions

HL carried out experiments; LY and ZZ carried out data analysis and drew figures; JM and WC wrote the paper; ET edited the paper; JM and GL led the research.

## Funding

This work was supported by the National Natural Science Foundation of China (81860629, 81471809), Major Project of Guangxi Science and Technology Department (AA17292001) and the Open Funds of the Guangxi Key Laboratory of Tumor Immunology and Microenvironmental Regulation (2018KF003). Project of Guangxi Medical and Health Self-financing Plan (Z20180420).

## Conflict of Interest Statement

The authors declare that the research was conducted in the absence of any commercial or financial relationships that could be construed as a potential conflict of interest.
